# A TELEWELLNESS APPROACH TO REDUCE BARRIERS TO GROUP EXERCISE FOR ADULTS AGING WITH MOBILITY DISABILITY

**DOI:** 10.1093/geroni/igz038.2788

**Published:** 2019-11-08

**Authors:** Tracy L Mitzner, Elena T Remillard

**Affiliations:** Georgia Institute of Technology, Atlanta, Georgia, United States

## Abstract

Group exercise classes have the potential to provide physical, cognitive, and emotional health benefits, through the physical activity performed as well as the social interaction among class participants. Substantial barriers exist for adults aging with lower-body mobility disabilities to engage in group exercise classes, including lack of transportation to classes, inaccessible buildings where classes are held, and lack of appropriate modifications offered for this population of older adults. Just as telehealth interventions have reduced barriers to healthcare, telewellness interventions can reduce barriers to engaging in wellness activities, such as group exercise classes. We will discuss a research study employing teleconferencing software to translate an evidence-based group tai chi class for adults aging with lower-body mobility disabilities. We will present the adaptation requirements identified to test the efficacy of a telewellness intervention for improving increasing social interaction and positive health behaviors (i.e., physical exercise frequency) for adults aging with disabilities.

